# Diseases of the Eye and Ophthalmoscopy

**Published:** 1897-09

**Authors:** 


DISEASES OF THE EYE AND OPHTHALMOSCOPY. A handbook for physicians and students. By A. EUGEN FICK, M. D., University of Zurich. Authorised translation by Albert B. Hale, A. B., M. D., one of the Ophthalmic Surgeons to the United Hebrew Charities; Consulting Ophthalmic Surgeon to Charity Hospital, Chicago. Octavo, pp. xvi.—488. With a glossary and 158 illustrations. Philadelphia: P. Blakiston, Son & Co., 1012 Walnut street. 1896.

While it can hardly be said that there is a real need of another "handbook" on diseases of the eye, even to represent German practice, yet this work of Dr. Fick's is a good one of its kind and will find many admirers. It covers the usual ground, omitting pathological hypotheses and details and references to authorities. The methods of examinations are described in considerable detail and constitute a most important part of the book. They include the function-tests for acuteness of vision, refraction, accommodation, light-sense, color-sense, field of vision, binocular vision and squint, and the objective methods of examination by Keratoscopy, focal or oblique illumination, ophthalmoscopy, skiascopy and palpation. Then follows a clear, concise and systematic description of the various diseases of the eye and its appendages and their treatment, together with the operations performed. Ocular therapeutics are up-to-date from the German standpoint.

The work is well illustrated and the colored engravings add materially to the elucidation of the text. The translator is to be congratulated on his success in rendering it into so good English.

Those who desire to acquaint themselves with the principles of German ophthalmology will find them well stated in this handbook of Dr. Fick. A. A. H.

